# Identification of hub genes and potential molecular mechanisms in MSS/MSI classifier primary colorectal cancer based on multiple datasets

**DOI:** 10.1007/s12672-024-01148-0

**Published:** 2024-07-18

**Authors:** Xia Qiao, Duan Ma, Xu Zhang

**Affiliations:** Institute of Medical Science, General Hospital of Ningxia Medical University, Yinchuan, 750004 China

**Keywords:** Colorectal cancer, Microsatellite instability, Microsatellite stability, Biomarkers, Immune checkpoint inhibitors

## Abstract

**Objective:**

MSI has a better prognosis than MSS in colorectal cancer patients, and the main objective of this study was to screen for differentially expressed molecules between MSI and MSS primary colorectal cancers using bioinformatics.

**Material and methods:**

Two gene expression datasets (GSE13294 and GSE13067) were downloaded from GEO, and differential expressed genes (DEGs) were analyzed using GEO2R. Gene Ontology, Kyoto Encyclopedia of Genomes, and Gene Set Enrichment Analysis were conducted using the DEGs. Furthermore, a Protein–Protein Interaction Networks (PPI) was constructed to screen for significant modules and identify hub genes. The hub genes were analyzed in colorectal cancer using GEPIA. The expression of hub genes in clinical samples was visualized using the online Human Protein Atlas (HPA).

**Results:**

A total of 265 common DEGs were identified in MSS primary colorectal cancer compared to MSI primary colorectal cancer. Among these, 178 DEGs were upregulated, and 87 DEGs were downregulated. Enrichment analysis showed that these DEGs were associated with the response to mechanical stimulus, regulation of cellular response to stress, G protein-coupled receptor binding, and other processes. A total of 5 hub genes was identified by cytoHubba: HNRNPL, RBM39, HNRNPH1, TRA2A, SRSF6. GEPIA software online analysis, 5 hub gene expression in colorectal cancer survival curve did not have significant differences. The expression of RBM39 was significantly different in different stages of colorectal cancer. The HPA online database results showed that the expression of the five hub proteins varied widely in CRC patients.

**Conclusion:**

The hub genes, such as HNRNPH1and RBM39, and the spliceosome resulting from DEGs, which may provide novel insights and evidence for the future diagnosis and targeted therapy of MSS/MSI PCRC.

**Supplementary Information:**

The online version contains supplementary material available at 10.1007/s12672-024-01148-0.

## Introduction

Primary colorectal carcinoma (PCRC) is a malignant tumor originating anywhere in the colon or rectum. PCRC has the third highest incidence rate and the fifth highest mortality rate among malignant tumors in China [1]. At present, the treatment of PCRC is mainly based on surgery, supplemented by radiotherapy, chemotherapy, targeted therapy and other comprehensive treatment, but some patients with middle and advanced colorectal cancer have a poor prognosis [2]. Previous studies have reported 5-year survival rates of up to 90% after early radical surgery, whereas CRC diagnosed as metastatic has a survival rate of less than 20% over 5 years [3, 4].

Cancer immunotherapy is an effective form of tumor treatment that overcomes the problem of specificity compared to standard treatments. Patients who respond well to cancer immunotherapy have prognosis and a better quality of life [5]. However, the efficiency of immune checkpoint inhibitors (ICIs) treatment is influenced by the microsatellite instability (MSI) status in each CRC patient [6]. Microsatellite instability (MSI), caused by deletion of DNA mismatch repair (MMR) protein expression or mutation of coding genes is associated with tumor development, and about 15% of colorectal cancers are associated with MSI [7]. Studies show that the PD-1 inhibitor Pembrolizumab is significantly more effective in treating MSI tumors than MSS tumors [8]. Studies have shown that in colorectal cancer patients, Pembrolizumab shows significant benefit to the mismatch-repair deficient or microsatellite instability-high CRC patients (dMMR/MSI-H) [9].Therefore, screening and analysis of molecular subtype-specific expressed genes of MSI and MSS may identify new diagnostic and therapeutic targets and provide a basis for the improvement of tumor immunotherapy sensitivity. Previous studies have explored a several genes and molecules participating in the tumor process as biomarkers, but little is known about the biomarkers applied to clinical MSI states [10].

Bioinformatics analysis and gene expression profiling by DNA microarrays have been extensively used to filter genetic alterations at the tumor genome level, helping us identify differentially expressed genes and functional pathways involved in the occurrence and progression of MSI CRC [11]. However, the false-positive rate in gene expression profiling of stand-alone DNA microarrays makes it difficult to obtain reliable results.

In this study, two mRNA microarray datasets were downloaded from the public database GEO and reanalyzed using GEO2R to obtain DEGs between MSI and MSS CRC. Then, GO and KEGG pathway enrichment analysis and PPI network analysis of DEGs were performed using GSEA for hub gene identification. Finally, we used the GEPIA database to validate the correlation of hub gene expression levels with prognostic correlations and in the development of PCRC to improve our understanding of MSI and MSS pathways in tumorigenesis and to provide information about potential biomarkers and therapeutic targets for PCRC. The results will provide a basis for the study of the mechanism of the 5 genes in the occurrence and development of PCRC and provide ideas for the application of five gene in the prevention and treatment of PCRC.

## Methods

### Microarray data

The Gene Expression Omnibus (GEO) (https://www.ncbi.nlm.nih.gov/geo/) data is an international public repository of high-throughput gene expression and other functional genomics data sets [12]. GSE13294 and GSE13067 were downloaded which were generated by HG-U133Plus 2.0 Affymetrix Human Genome arrays (Platform GPL570) [13]. A total of 229 samples were analyzed, consisting of 140 microsatellite stable samples and 89 microsatellite instability samples taken from colorectal cancer patients.

### Screening of the DGEs

DEGs (Differential Expressed Genes) were performed by GEO2R (https://www.ncbi.nlm.nih.gov/geo/geo2r/#). Two groups of sample chips were created in GEO2R, namely MSS PCRC samples and MSI PCRC samples, and all differential genes were screened by selecting the save all results option. To obtain adjusted p-values (adj. P), we corrected the p-values using the Benjamini and Hochberg technique (false discovery rate) to achieved a trade-off between the possibility of false-positive results and the identification of statistically significant genes. To improve the screening efficiency, candidate DEGs were screened with the filter criteria: P value < 0.05, logarithmic change (logFC) ≥ 1 or (logFC) ≤ − 1.

### GO and KEGG enrichment analysis of DEGs

The biological processes, cellular components, and molecular functions of DEGs were annotated through the Gene Ontology (GO) Knowledge Base (http://geneontology.org) and the genecards database (https://www.genecards.org/). The Kyoto Encyclopedia of Genes and Genomes (KEGG, https://www.kegg.jp) analysis was performed to determine the target genes in biological pathway [14]. GO annotation and KEGG pathway functional annotation were conducted for DEGs using the online tool DAVID (DAVID Functional Annotation Bioinformatics Microarray Analysis (ncifcrf.gov)) [15]. Pathway and cellular process enrichment analyses were applied using Metascape to allow experimental biologists to comprehensively analyze and interpret OMICs-based studies in the era of big data [16]. Gene set enrichment analysis (GSEA) was used to elucidate significant functional and pathway differences between MSI and MSS CRC group [17].

### PPI network construction, significant module, and hub genes network

To further explore the interactions between DEGs in this study, the protein–protein interaction network of DEGs was identified using the Search Tool for the Retrieval of Interacting Genes/Proteins (STRING, PPI, https://string-db.org/) [18]. In this study, interactions with a composite score of > 0.4 were considered statistically significant. Cytoscape (version 3.9.1) (https://cytoscape.org) software was used for analysis and visualization of the PPI network. Applying MCODE (version 2.0.2) to identify the most significant modules in a PPI network [19]. The selection criteria are as follows: degree cut-off = 2, node score cut-off = 0.2, Max depth = 100 and k-score = 2. Then, the top 5 genes were selected and identified as hub genes by MCC topology analysis of cytoHubba.

### ***Hub genes selection and analysis***

The top 5 genes were selected and identified as hub genes by MCC topology analysis of cytoHubba. Hierarchical clustering of hub genes was constructed using UCSC Cancer Genomics Browser (UCSC Xena (xenabrowser.net)) [20]. The overall survival and disease-free survival analyses of hub genes were performed using Kaplan–Meier curve in Gene Expression Profiling Interactive Analysis (GEPIA). Additionally, TCGA data was used for validation using the GEPIA database.

### *Validation of the protein expression levels of the hub genes *via* the human protein atlas*

To further verify the protein expression levels of the five hub genes in colorectal cancer and normal tissues, immunohistochemistry (IHC) data were downloaded from the Human Protein Atlas (HPA) (http://www.proteinatlas.org). HPA canprovide IHC results for a variety of proteins based on proteomics in cancer tissues and normal tissues.

### Statistical analysis

Cox regression analysis was used to screen out prognostic genes. Survival analysis was performed by Kaplan–Meier method. Comparison between groups was performed by Wilcox test. P < 0.05 was considered statistically significant.

## Results

### Identification of DEGs in MSS/MSI PCRC

**Fig. 1:**
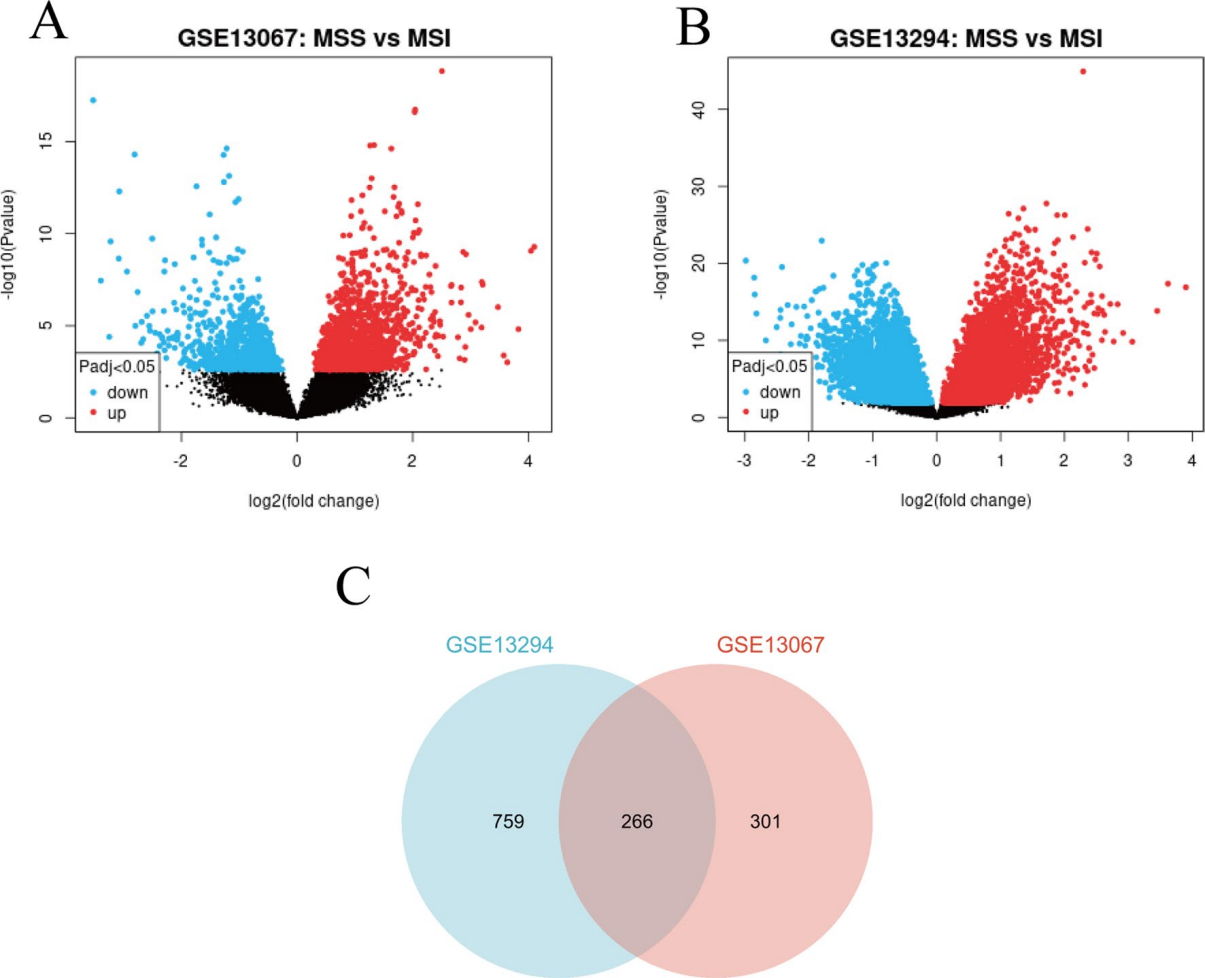
Differentially expressed genes. **A** volcano plot of DEGs in the GSE13067 dataset. Black dots indicate no differential expression between MSS CRCs and MSI CRCs. Blue dots represent downregulated genes, and red dots represent upregulated genes. **B** volcano plot of DEGs in the GSE13294 dataset. Black dots indicate no differential expression between MSS CRCs and MSI CRCs. Blue dots represent downregulated genes, and red dots represent upregulated genes **C** Venn diagram of the DEGs in GSE13294 and GSE13067

With the screening criteria of P < 0.001, (logFC) ≥ 1 or ≤ -1(GEO2R analysis), 1552 DEGs in GSE13294 and 833 DEGs GSE13067 were identified (Fig. 1A and B). (The overlap among the 2 datasets contained 265 genes as shown in Venn diagram (Fig. 1C), consisting of 178 upregulated genes and 87 downregulated genes between MSS Primary colorectal carcinoma and MSI Primary colorectal carcinoma patients.

### *KEGG and GO enrichment analyses of DEGs *via* DAVID and metascape*

**Fig. 2:**
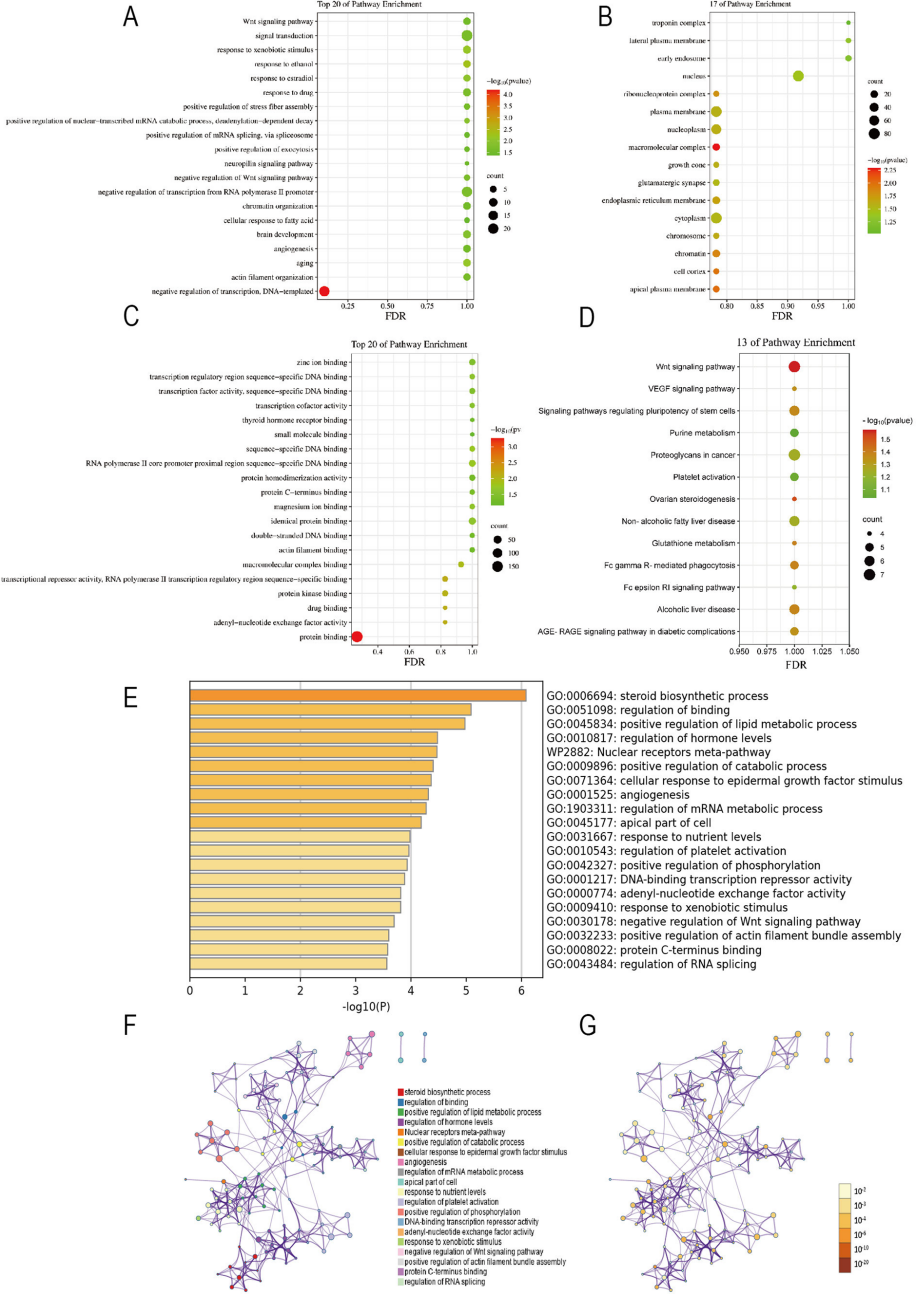
The enrichment analysis of DEGs by DAVID and Metascape. **A** Biological process for the top 20 pathway enrichment. **B** Cell component for the top 17 pathway enrichment **C** Molecular function for the top 20 pathway enrichment. **D** KEGG enrichment for the top 13 pathway enrichment. **E** Heatmap of enriched terms across input differentially expressed gene lists, colored by p-values, via Metascape. **F** Network of enriched terms colored by cluster identity, where nodes that share the same cluster identity are typically close to each other. **G** Network of enriched terms colored by p-value, where terms containing more genes tend to have a more significant p-value

DAVID analysis results showed variations in DEGs associated with biological processes (BP) were mainly enriched in the negative regulation of transcription, DNA-templated, actin filament organization, aging, angiogenesis, brain development, and other processes (Fig. 2A). Variations in DEGs associated with cell component (CC) were mainly enriched in the apical plasma membrane, cell cortex, chromatin, cytoplasm, endoplasmic reticulum membrane, etc. (Fig. 2B). Variations in DEGs associated with molecular function (MF) were mainly enriched in protein binding, adenyl-nucleotide factor activity, drug binding, protein kinase binding, transcriptional repressor activity, RNA polymerase II transcription regulatory region sequence-specific binding, macromolecular complex binding, etc. (Fig. 2C). KEGG pathway analysis indicated that DEGs were mainly enriched in the Wnt signaling pathway, AGE-RAGE signaling pathway in diabetic complications, alcoholic liver disease, Fc epsilon RI signaling pathway, Fc gamma R-mediated phagocytosis, glutathione metabolism, non-alcoholic fatty liver disease, and ovarian steroidogenesis, etc. (Fig. 2D).

Moreover, Metascape analysis showed that functional enrichment of the DEGs was significantly enriched in steroid biosynthetic process, regulation of binding, positive regulation of lipid metabolic process, regulation of hormone levels, Nuclear receptors meta-pathway, and positive regulation of catabolic process, etc.( P < 0.05, Fig. 2E-G).

### GO and KEGG pathway enrichment analysis of DEGs in PCRC using GSEA

**Fig. 3:**
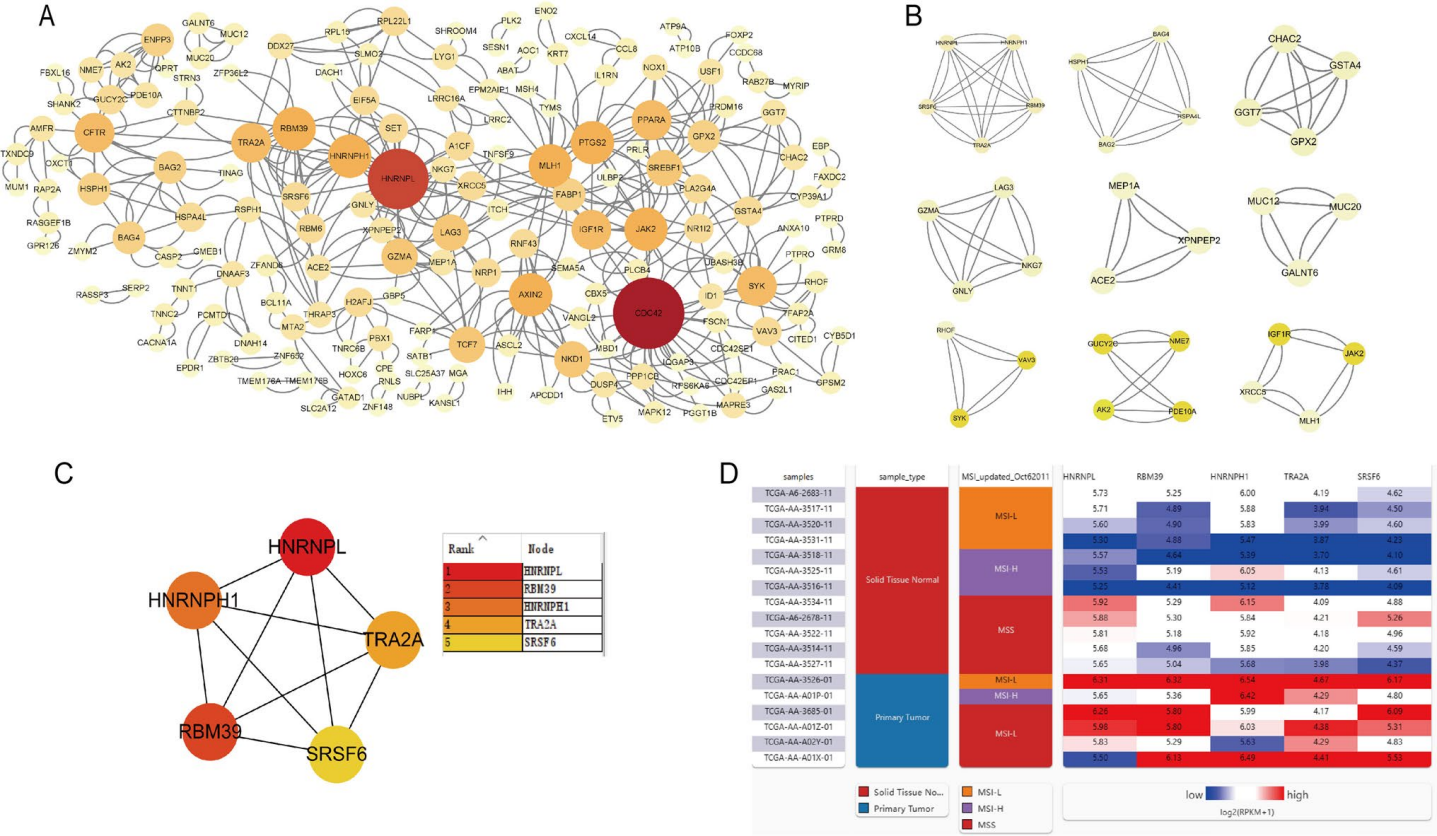
PPI network and hub gene. **A** Protein–protein interaction network of the differently expression genes between MSS primary tumor samples and MSI primary tumor samples. **B** A total of 9 significant modules identified from the protein–protein interaction network. **C** Top 5 core gene interaction networks, with darker colors indicating a higher Node of critical importance. **D** Hierarchical clustering of hub genes was constructed using UCSC. Highly expressed genes are marked in red; Lowly expressed genes are marked in blue

**Fig. 4:**
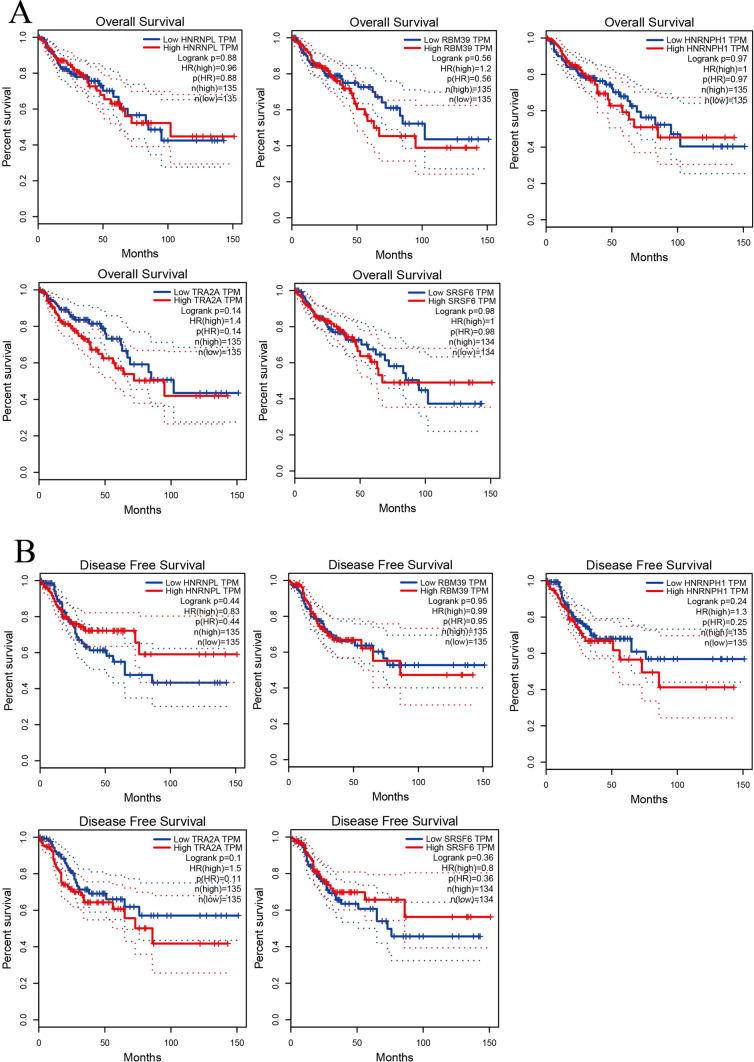
Survival analysis of 5 candidate genes. **A** Overall survival. **B** Disease Free Survival analyses of hub genes performed using GEPIA online platform. P < 0.05 was considered statistically significant

Through GSEA analysis, the results of GO and KEGG analysis were used to explore the significantly enriched pathways of DEGs. GO enrichment analysis revealed that 183 upregulated gene sets were significantly enriched with a P-value < 0.05. Additionally, 71 downregulated gene sets were significantly enriched with a P-value < 0.05. The most significant upregulation and downregulated gene enrichments are listed in Table 1, which includes the top ten GO gene sets based on the NES. GO enrichment analysis results showed that upregulated gene sets in PCRC are mainly associated with the response to mechanical stimulus, regulation of cellular response to stress, G protein-coupled receptor binding, sulfur compound metabolic process, and positive regulation of neuron projection development. On the other hand, downregulated gene sets in PCRC are mainly associated with neural retina development, embryonic camera-type eye development, embryonic eye morphogenesis, embryonic camera-type eye morphogenesis, and cellular component assembly involved in morphogenesis. Six significant enrichment plots are shown in Fig s. 1 and 2. Furthermore, KEGG pathway enrichment analysis results showed that upregulated gene sets in PCRC are mainly associated with the pathways of adherens junction, endocytosis, axon guidance, fc gamma r mediated phagocytosis, prostate cancer (Table 2). Four significant enrichment plots are shown in Figs. 3 and 4. Besides, downregulated gene set in PCRC is mainly associated with the pathways of spliceosome, nod like receptor signaling pathway, leishmania infection, glycolysis gluconeogenesis, RNA degradation (Table 2).

**Table 1:** Functional enrichment analysis of DEGs in PCRC using GSEA

Gene Set Name	SIZE	ES	NES	P-value
Upregulated				
GOBP_RESPONSE_TO_MECHANICAL_STIMULUS	4	0.92	1.76	0.000
GOBP_REGULATION_OF_CELLULAR_RESPONSE_TO_STRESS	9	0.69	1.72	0.006
GOMF_G_PROTEIN_COUPLED_RECEPTOR_BINDING	6	0.73	1.68	0.015
GOBP_SULFUR_COMPOUND_METABOLIC_PROCESS	6	0.72	1.62	0.025
GOBP_POSITIVE_REGULATION_OF_NEURON_PROJECTION_DEVELOPMENT	3	0.96	1.62	0.002
Downregulated				
GOBP_NEURAL_RETINA_DEVELOPMENT	3	− 0.86	− 1.52	0.029
GOBP_EMBRYONIC_CAMERA_TYPE_EYE_DEVELOPMENT	2	− 0.88	− 1.44	0.056
GOBP_EMBRYONIC_EYE_MORPHOGENESIS	2	− 0.88	− 1.44	0.056
GOBP_EMBRYONIC_CAMERA_TYPE_EYE_MORPHOGENESIS	2	− 0.88	− 1.44	0.056
GOBP_CELLULAR_COMPONENT_ASSEMBLY_INVOLVED_IN_MORPHOGENESIS	2	− 0.90	− 1.43	0.044

PCRC: primary colorectal cancer; ES: Enrichment Score; NES: Normalized Enrichment Score

**Table 2:** Pathway enrichment analysis of DEGs in PCRC using GSEA

Gene Set Name	SIZE	ES	NES	P-value
Upregulated				
KEGG_ADHERENS_JUNCTION	3	0.90	1.64	0.002
KEGG_ENDOCYTOSIS	3	0.93	1.57	0.004
KEGG_AXON_GUIDANCE	3	0.85	1.54	0.020
KEGG_FC_GAMMA_R_MEDIATED_PHAGOCYTOSIS	5	0.71	1.50	0.034
KEGG_PROSTATE_CANCER	2	0.92	1.46	0.028
Downregulated				
KEGG_SPLICEOSOME	2	− 0.90	− 1.40	0.040
KEGG_NOD_LIKE_RECEPTOR_SIGNALING_PATHWAY	2	− 0.90	− 1.31	0.080
KEGG_LEISHMANIA_INFECTION	3	− 0.80	− 1.27	0.170
KEGG_GLYCOLYSIS_GLUCONEOGENESIS	1	− 0.98	− 1.25	0.037
KEGG_RNA_DEGRADATION	1	− 0.98	− 1.25	0.037

PCRC: primary colorectal cancer; ES: Enrichment Score; NES: Normalized Enrichment Score

### PPI construction, module analysis, and identification of hub genes

Through STRING analysis, the *P*-value of the PPI was < 0.05, and the network consisted of 236 nodes and 228 edges (Fig. 3A). A total of 9 significant modules were identified with MCODE from the PPI network (Fig. 3B). A total of 5 hub genes were identified based on the Node score of cytoHubba: HNRNPL, RBM39, HNRNPH1, TRA2A, and SRSF6 (Fig. 3C). Subsequently, Hierarchical clustering revealed that the hub genes essentially distinguished MSS CRC samples from MSI CRC samples (Fig. 3D).

### Hub gene selection and analysis

**Fig. 5:**
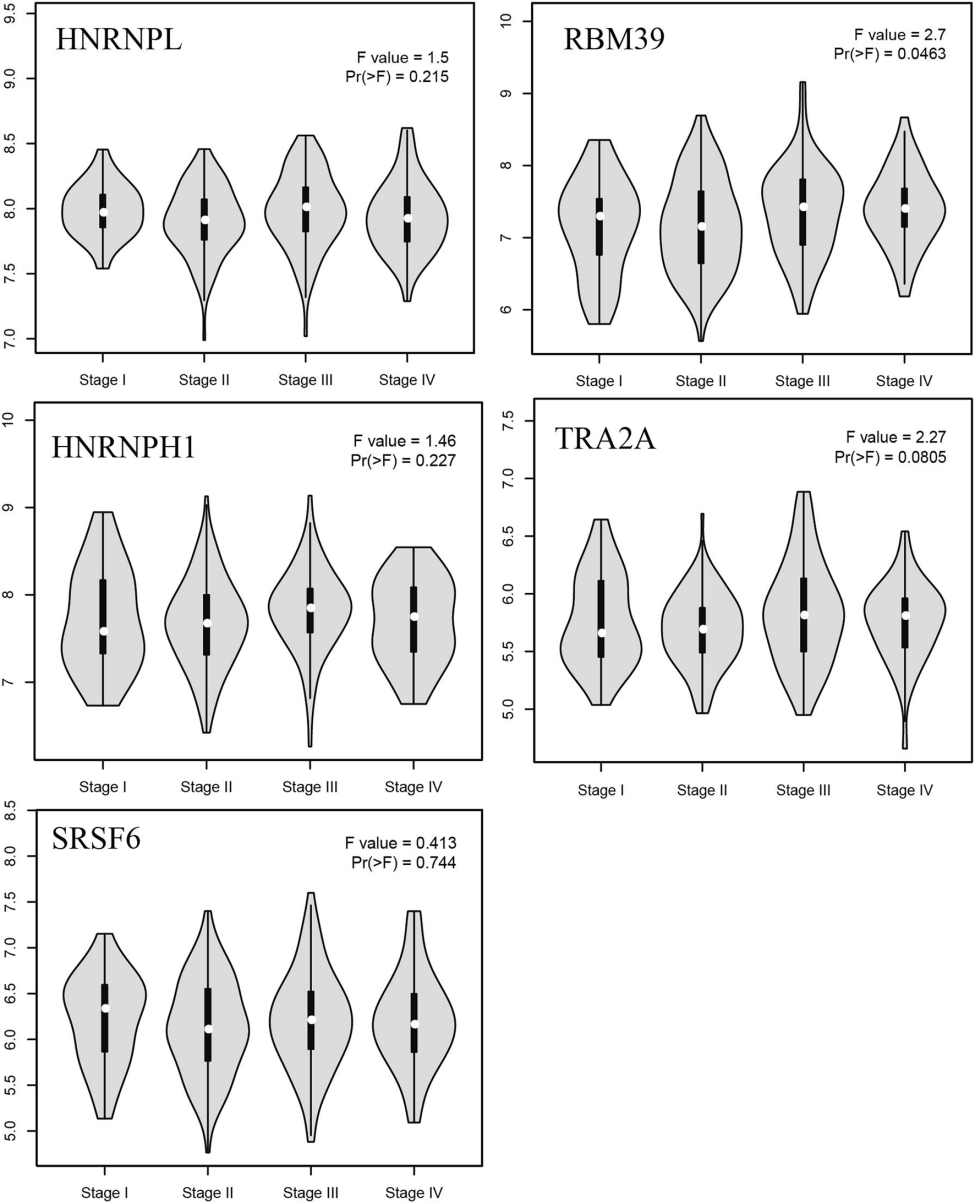
Stage plot of the hub genes. P < 0.05 was considered statistically significant

A total of 5 genes were identified as hub genes. The name, abbreviations and functions for these hub genes are shown in Table 3. Subsequently, the overall survival analysis of the hub genes was performed using Kaplan–Meier curves. The hub genes did not differ significantly in overall survival and disease-free survival (Fig. 4). The expression of hub genes in different stages of CRC was analyzed by stage plot in GEPIA. RBM39 gene has significant differences in CRC development (Fig. 5).

**Table 3:** Functinal roles of 5 hub genes

No	Gene symbol	Full name	Function	References
1	HNRNPL	Heterogeneous Nuclear Ribonucleoprotein L	HNRNPL promotes the tumor growth and development of CRC by regulating PD-L1	[21]
2	RBM39	RNA-binding motif protein 39	RBM39 is the unexpected target of aryl sulphonamides	[22]
3	HNRNPH1	Heterogeneous Nuclear Ribonucleoprotein H1	serum exosomal hnRNPH1 mRNA could be an effective marker for HCC in high HBV prevalence areas	[23]
4	TRA2A	Transformer 2 Alpha Homolog	TRA2A facilitates proliferation and survival and migration and invasion of TNBC cells	[24]
5	SRSF6	TNF Receptor Associated Factor 6	TRAF6 plays a critical role in lymphatic metastasis of colorectal cancer	[25]

### Validation of the expression levels of five hub genes in clinical samples

**Fig. 6:**
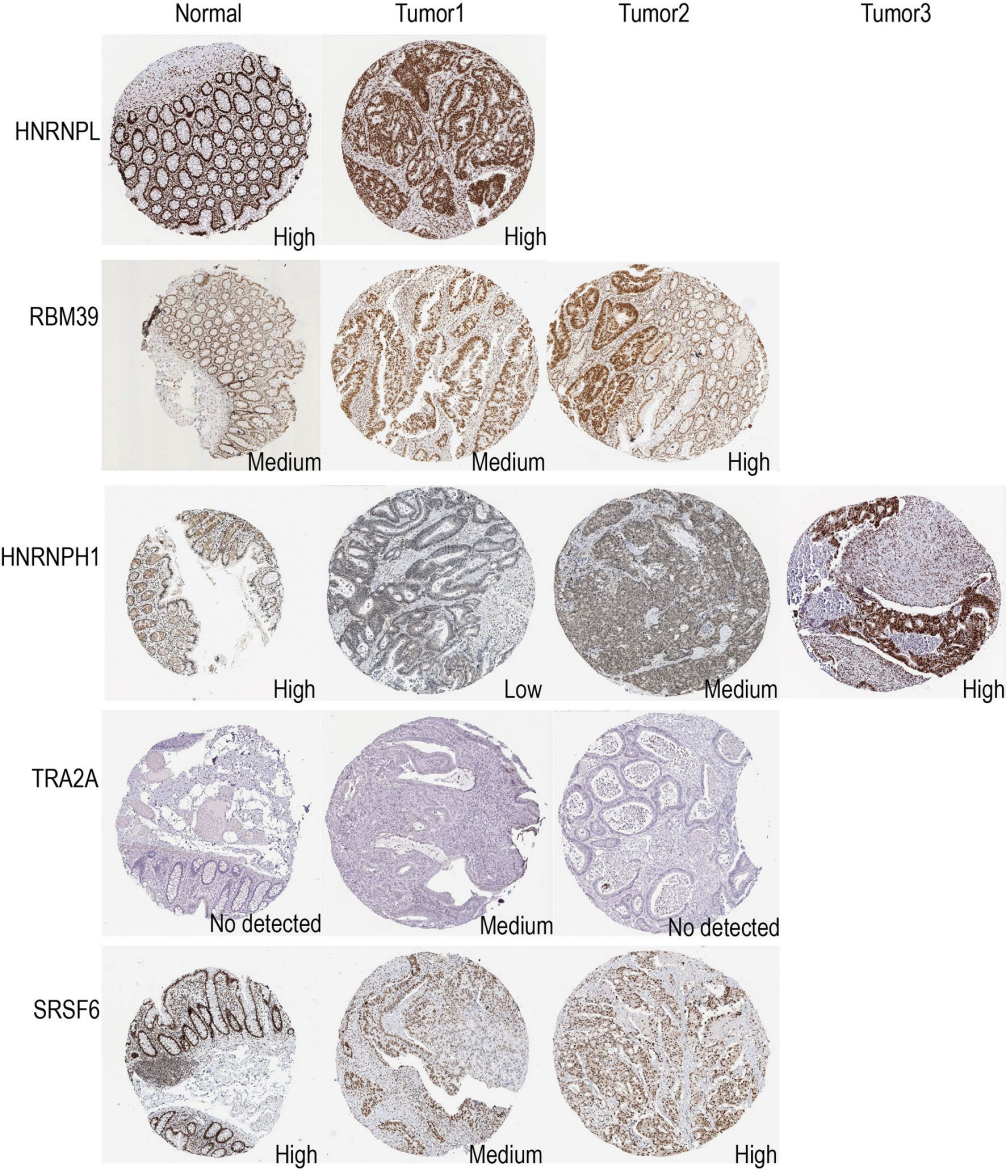
The protein expression levels of HNRNPL, RBM39, HNRNPH1, TRA2A and SRSF6 in normal colorectal tissues and colorectal cancers from HPA online database

IHC results of the protein expression of HNRNPL、RBM39、HNRNPH1、TRA2A and SRSF6 from the HPA database are displayed in Fig. 6. HNRNPL expression levels were high in both normal colorectal and colorectal cancer tissues. The expression level of RBM39 in normal colorectal tissues was moderate, and it showed moderate to high expression in colorectal cancer patients. The expression level of HNRNPH1 was high in normal colorectal tissues and varied from low to high expression in colorectal cancer patients. SRSF6 was highly expressed in normal colorectal tissues and had medium to high expression levels in CRC patients. Moreover, the protein expression level of TRA2A was not detected in normal colorectal tissues, and was moderately expressed in CRC patients. The different expression levels of these five hub genes may explain the differences in intrinsic biological characteristics and individual differences in the sensitivity of CRC patients to immunotherapy.

## Discussion

CRC remains one of the leading causes of cancer deaths worldwide and is a heterogeneous disease associated with many genetic or somatic mutations. Primary colorectal cancer and metastatic colorectal cancer are treated differently and require individualized treatment [26, 27]. Most patients with early-detected CRC have high survival, while the 5-year survival rate is about 12.5% for patients with advanced metastatic CRC [28]. MSI has a better prognosis in early CRC and a worse prognosis in metastasis [29]. In addition, MSI-H is particularly responsive to immunotherapy and is considered a promising subtype associated with CRC immunotherapy. However, its pathogenesis and complexity are not yet fully understood. In particular, there is a lack of clarity on the potential pathogenesis and associated targets of MSI CRC. Therefore, there is an urgent need for potential markers for efficient diagnosis and therapy.

In this study, 2 mRNA microarray datasets were analyzed to obtain DEGs between MSS PCRC and MSI PCRC. A total of 265 DEGs were identified, of which 178 genes were upregulated and 87 genes were downregulated. With GO and KEGG enrichment analyses, we found that the DEGs were mainly involved in metabolism and DNA repair. Interestingly, we found that the WNT signaling pathway was significantly between the MSI and MSS PCRC groups through KEGG pathway analysis. Previous studies have shown that the increase of Wnt/β-catenin activity in colorectal cancer was related to the lack of T cell infiltration in the tumor microenvironment [30, 31], and Wnt/β-catenin signaling may play an important role in the process of “immune exclusion”, providing new ideas for the effectiveness of immunotherapy. The relationship between these hub genes and others signaling pathways in this study in immunotherapy requires further investigation. Further discussion on hub genes.

Heterogeneous nuclear ribonucleoprotein L (hnRNPL), as a splicing regulatory protein, is involved in many important biological processes, including DNA repair, RNA alternative splicing, transcription factor activity, and protein translation [32–34]. In addition, HNRNL plays an important role in the development of many cancers [35, 36]. HNRNRL can act as a p53 mRNA binding protein and play an important role in the proliferation and apoptosis of nephroblastoma through the p53 and Bcl2 pathways [37]. Degradation of HNRNRL inhibits ovarian cancer cell growth in vitro and in vivo, and additionally makes esophageal squamous cell carcinoma chemoresistant [38, 39]. In a recent study, HNRNPL was identified as a prognostic biomarker associated with microsatellite instability in gastric cancer, suggesting that it may be a new target for the treatment of MSI gastric cancer [40]. In this study, HNRNRL was found to be highly expressed in MSI PCRC by informatics, but the survival curves were not significant in CRC compared with normal tissues, so we speculated that HNRNRL might be a prognostic biomarker for MSI PCRC, but the mechanism is not clear and needs further verification.

RNA‐binding Motif Protein39 (RBM39) is known as a splicing factor and transcription coactivator. As an essential mRNA splicing factor and potential target for cancer therapy, studies have that the oncogenic agent indisulam inhibits cell proliferation by causing degradation of RBM39 [28, 41, 42].Furthermore, Enhanced checkpoint immunotherapy through pharmacological modulation of splicing by specific drug classes can generate true neoantigens and trigger anti-tumor immunity [43]. Previous studies have also shown that RBM39 is a proto-oncogene that plays an important role in the development of malignant tumors [44]. Recent studies have found that RBM39 has been identified as a marker of pan-cancer and is negatively correlated with the infiltration of most immune cells [45]. In particular, RBM39 expression was substantially associated with tumor stage and MSI, which is similar to the findings of this study.

In addition, three other proteins analyzed in this study, HNRNPH1, TRA2A, and SRSF6, were found to be involved in cancer development in previous studies [12, 13]. In particular, the splicing factor SRSF6 is recognized as an oncoprotein that regulates the proliferation and survival of lung and colon cancer cells [24]. The function of these hub genes in the functional mechanisms of CRC is poorly understood and needs to be further validated.

Despite the rigorous bioinformatics analysis in this study, there are still some drawbacks: (1) the sample size in the dataset is small and needs to be further expanded to obtain more accurate results. Nevertheless, significant DEGs were identified; (2) Current online database survival analyses are comparing CRC and normal tissues, so the hub genes identified in this study need to be comprehensively validated with a large number of clinical samples and animal experiments to gain insight into the molecular mechanisms of CRC development.

This study has several limitations. First, the data set is limited because they are according to the conditions of this study from a public database retrieval. Second, verify the number of samples is limited, because they are from a public database for verification. Finally, the hub genes were screened from the database, and additional basic experiments should be performed to investigate their molecular mechanisms and clinical specimens to verify their biological functions.

## Conclusions

In this study, the DEGs between MSI PCRC and MSS PCRC were identified by bioinformatics, and 265 DEGs and five hub genes were identified. Among these hub genes, HNRNPL and HNRNPH1 genes may be molecular markers for early colorectal cancer diagnosis in MSS.TRA2A and SRSF6 may play important roles in PCRC metastasis. RBM39 was significantly differentially expressed in PCRC at different stages. HNRNPL and HNRNPH1 are associated with CRC immunotherapy and may be potential targets for enhancing immune checkpoints. These results provide new insights into the diagnosis, treatment, and pathogenesis of MSS CRC.

### Supplementary Information

Additional file 1.

Additional file 2.

## Data Availability

Data is provided within the manuscript or supplementary information files. The bioinformation analysis datasets generated in the current study are available online as cited in the materials and methods.

## Abbreviations

GEO: Gene Expression Omnibus
GO: Gene Ontology
KEGG: Kyoto Encyclopedia of Genes and Genomes
GSEA: Gene set enrichment analysis
PPI: Protein–Protein Interaction Networks
MSS: Microsatellite stable
MSI: Microsatellite instability
ICIs: Immune checkpoint inhibitors
PCRC: Primary colorectal carcinoma
CRC: Colorectal cancer
MMR: Mismatch repair
DEGs: Differentially expressed genes
BP: Biological processes
CC: Cell component
MF: Molecular function
IHC: Immunohistochemistry
HPA: Human Protein Atlas
