# Supplementary material for: Identification of hub genes and potential molecular mechanisms in MSS/MSI classifier primary colorectal cancer based on multiple datasets
Source: Discov Oncol. 2024 Jul 18;15:290. doi: 10.1007/s12672-024-01148-0 (PMC11258107; doi:10.1007/s12672-024-01148-0)
Supplement: Supplementary file 2 — Additional file 2. [file 12672_2024_1148_MOESM2_ESM.docx]

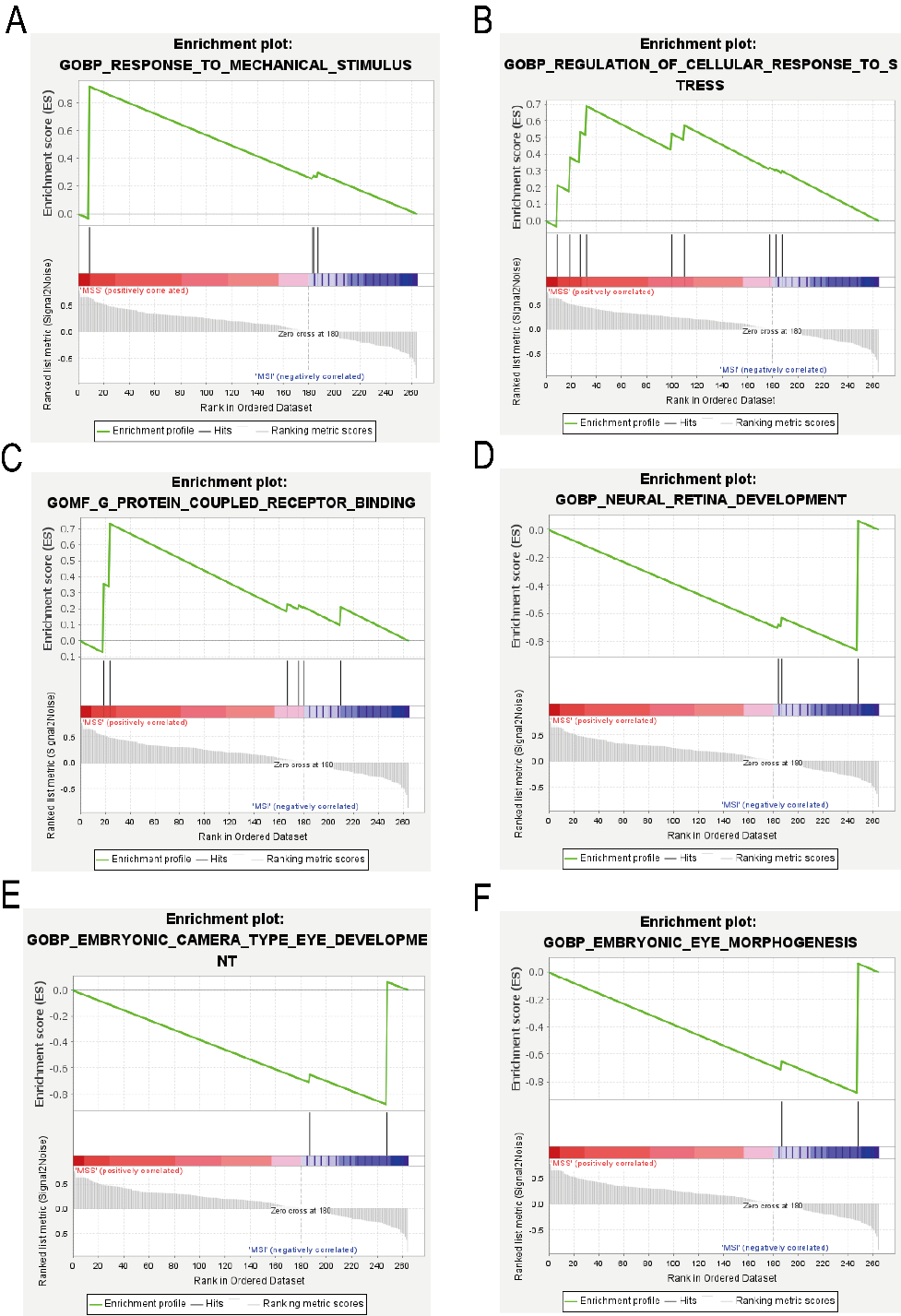


Figure S1: Six significant enrichment plots of functional enrichment analysis of DEGs in MSS PCRC and MSI PCRCR using GSEA. (A) Enrichment plot: GOBP_ RESPONSE_TO_MECHANICAL_STIMULUS. (B) Enrichment plot: GOBP_REGULATION_OF_CELLULAR_RESPONSE_TO_STRESS. (C) Enrichment plot: GOMF_G_PROTEIN_COUPLED_RECEPTOR_BINDING. (D) Enrichment plot: GOBP_NEURAL_RETINA_DEVELOPMENT. (E) Enrichment plot: GOBP_EMBRYONIC_CAMERA_TYPE_EYE_DEVELOPMENT. (F) Enrichment plot: GOBP_EMBRYONIC_EYE_MORPHOGENESIS


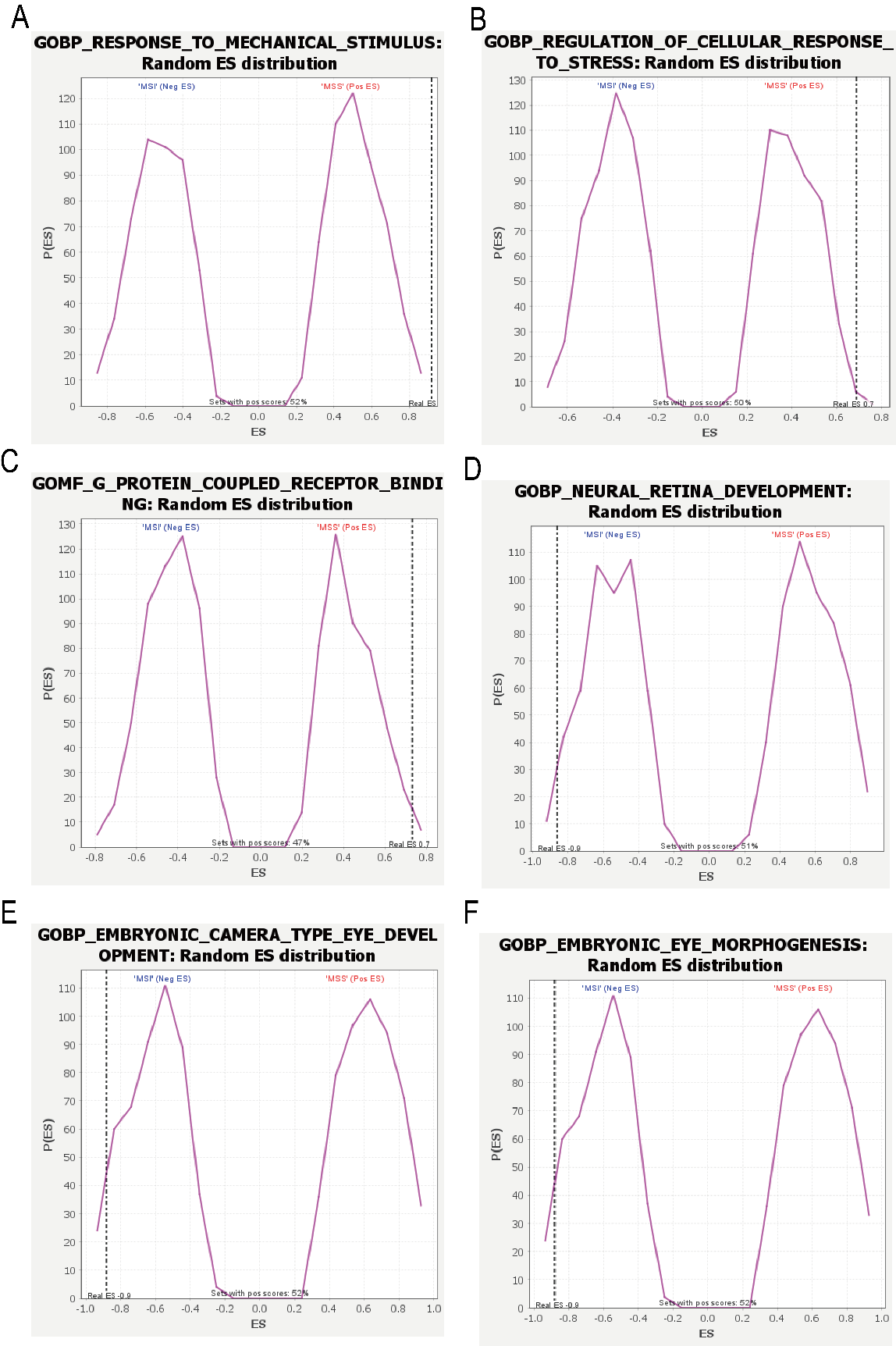


Figure S2: Random ES distribution of six significant enrichment plots. (A) RESPONSE_TO_MECHANICAL_STIMULUS: Random ES distribution. Gene set null distribution of ES for RESPONSE_TO_MECHANICAL_STIMULUS. (B) GOBP_REGULATION_OF_CELLULAR_RESPONSE_TO_STRESS: Random ES distribution. Gene set null distribution of ES for GOBP_REGULATION_OF_CELLULAR_RESPONSE_TO_STRESS. (C) GOMF_G_PROTEIN_COUPLED_RECEPTOR_BINDING: Random ES distribution. Gene set null distribution of ES for GOMF_G_PROTEIN_COUPLED_RECEPTOR_BINDING. (D) GOBP_NEURAL_RETINA_DEVELOPMENT: Random ES distribution. Gene set null distribution of ES for GOBP_NEURAL_RETINA_DEVELOPMENT. (E) GOBP_EMBRYONIC_CAMERA_TYPE_EYE_DEVELOPMENT: Random ES distribution. Gene set null distribution of ES for GOBP_EMBRYONIC_CAMERA_TYPE_EYE_DEVELOPMENT. (F) GOBP_EMBRYONIC_EYE_MORPHOGENESIS: Random ES distribution. Gene set null distribution of ES for GOBP_EMBRYONIC_EYE_MORPHOGENESIS


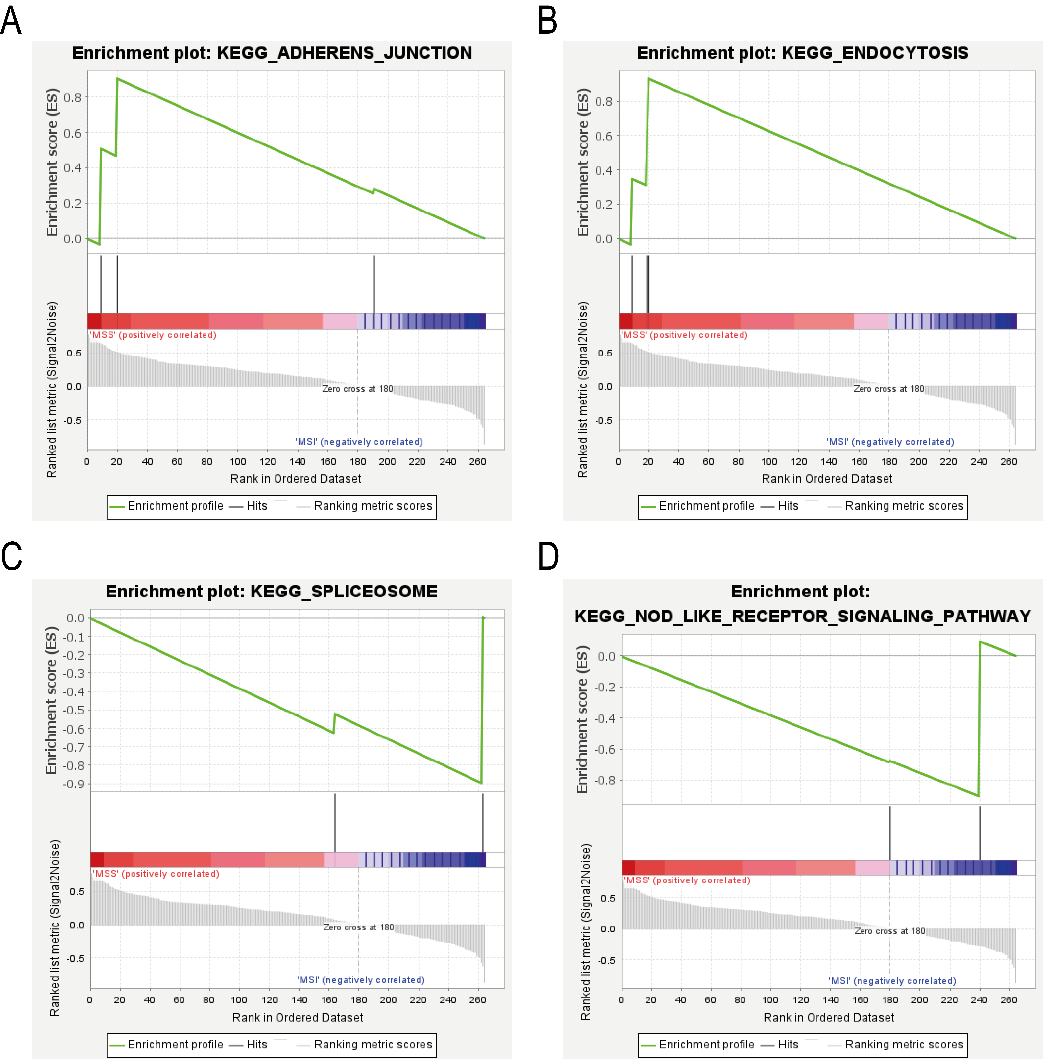


Figure S3: Four significant enrichment plots of pathway enrichment analysis of DEGs in MSS PCRC and MSI PCRC using GSEA. (A) Enrichment plot: KEGG_ADHERENS_JUNCTION. (B) Enrichment plot: KEGG_ENDOCYTOSIS. (C) Enrichment plot: KEGG_SPLICEOSOME. (D) Enrichment plot: KEGG_NOD_LIKE_RECEPTOR_SIGNALING_PATHWAY.


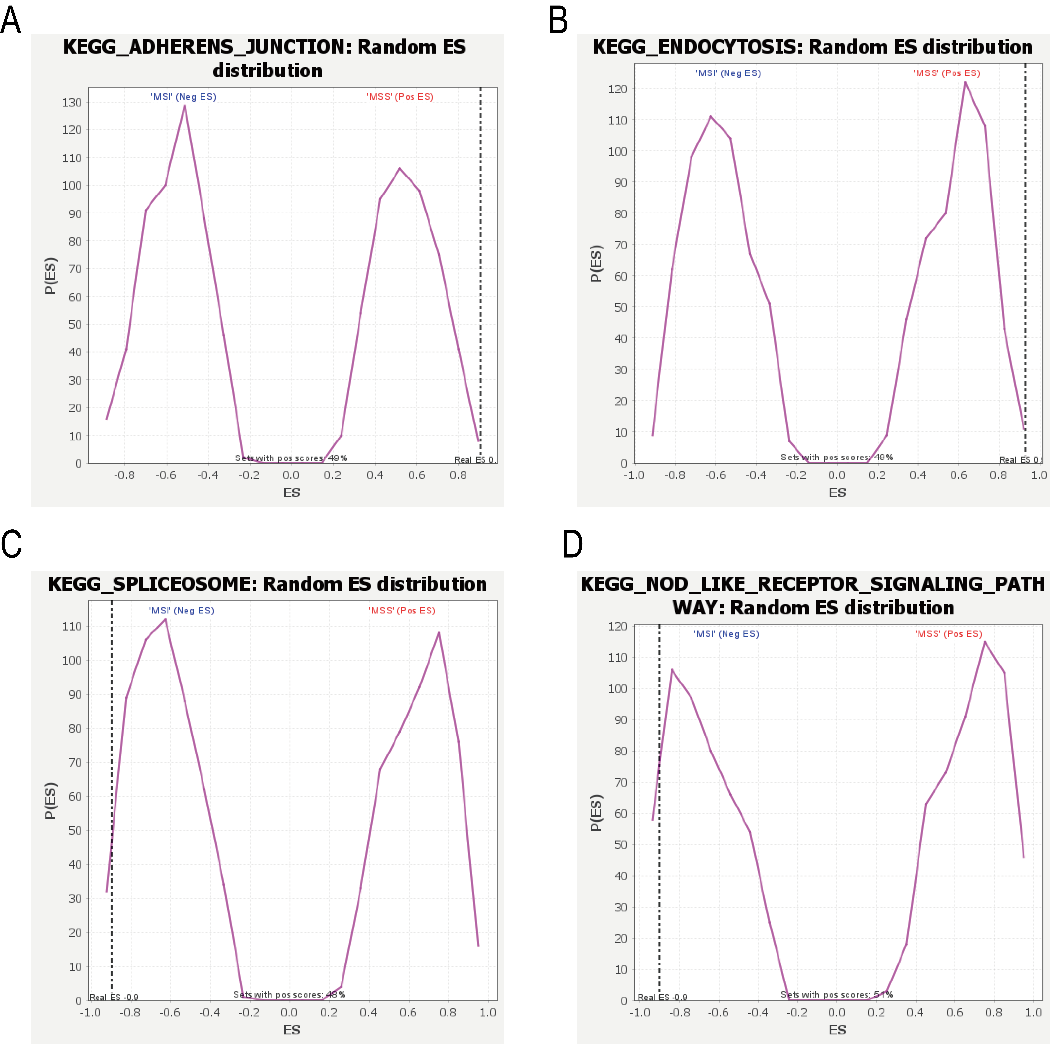


Figure S4: Random ES distribution of four significant enrichment plots of pathway enrichment analysis. (A) KEGG_ADHERENS_JUNCTION: Random ES distribution. Gene set null distribution of ES for KEGG_ADHERENS_JUNCTION. (B) KEGG_ENDOCYTOSIS: Random ES distribution. Gene set null distribution of ES for KEGG_ENDOCYTOSIS. (C) KEGG_SPLICEOSOME: Random ES distribution. Gene set null distribution of ES for KEGG_SPLICEOSOME. (D) KEGG_NOD_LIKE_RECEPTOR_SIGNALING_PATHWAY: Random ES distribution. Gene set null distribution of ES for KEGG_NOD_LIKE_RECEPTOR_SIGNALING_PATHWAY.
